# Nanomaterial-Based Fluorescence Resonance Energy Transfer (FRET) and Metal-Enhanced Fluorescence (MEF) to Detect Nucleic Acid in Cancer Diagnosis

**DOI:** 10.3390/biomedicines9080928

**Published:** 2021-07-31

**Authors:** Jin-Ha Choi, Taehyeong Ha, Minkyu Shin, Sang-Nam Lee, Jeong-Woo Choi

**Affiliations:** 1 School of Chemical Engineering, Jeonbuk National University, Jeonju 54896, Korea; jhchoi@jbnu.ac.kr; 2 Department of Chemical and Biomolecular Engineering, Sogang University, Seoul 04107, Korea; hatae91@sogang.ac.kr (T.H.); mkshin91@sogang.ac.kr (M.S.); 3 Uniance Gene Inc., 1107 Teilhard Hall, 35 Baekbeom-Ro, Mapo-Gu, Seoul 04107, Korea

**Keywords:** fluorescence resonance energy transfer, metal-enhanced fluorescence, nucleic acid, cancer, nanoparticle

## Abstract

Nucleic acids, including DNA and RNA, have received prodigious attention as potential biomarkers for precise and early diagnosis of cancers. However, due to their small quantity and instability in body fluids, precise and sensitive detection is highly important. Taking advantage of the ease-to-functionality and plasmonic effect of nanomaterials, fluorescence resonance energy transfer (FRET) and metal-enhanced fluorescence (MEF)-based biosensors have been developed for accurate and sensitive quantitation of cancer-related nucleic acids. This review summarizes the recent strategies and advances in recently developed nanomaterial-based FRET and MEF for biosensors for the detection of nucleic acids in cancer diagnosis. Challenges and opportunities in this field are also discussed. We anticipate that the FRET and MEF-based biosensors discussed in this review will provide valuable information for the sensitive detection of nucleic acids and early diagnosis of cancers.

## 1. Introduction

Cancer is one of the dominant and highly influential diseases in human life. Even though the five-year survival rates have gradually increased, the death rate is still above 0.1%, and the cancer incidence is approximately 0.4% [1,2]. In addition, cancer has become more frequent due to the increasing lifespan. For effective treatment, and to increase the cure rate of cancers, it is crucial to diagnose the exact types and stages precisely and sensitively. For example, some anticancer drugs are effective only against a certain type of cancer [3]. However, early diagnosis of cancers makes a recovery easier than late diagnosis due to the severe progression and metastasis of cancers [4,5]. Therefore, it is essential to develop a precise and sensitive diagnostic strategy that can detect and identify cancer tissue at the early stage and analyze its characteristics for the appropriate therapy. 

As cancer develops, several biological changes, such as cellular levels of DNA, RNA, protein, and other small molecules, occur in body fluids [6,7,8,9]. Thus, quantitation of these molecules can be used as an essential parameter for cancer diagnosis. Among them, nucleic acids have been used as potential biomarkers due to their differential levels between cancer patients and healthy individuals [10,11]. Compared with other biomarkers, nucleic acids have the benefit and potential of serving as relatively stable biomarkers in body fluids for cancer diagnosis. However, nucleic acids have some limitations as potential biomarkers. A major challenge of using them is the low concentration of target sequences in the presence of high noise factors, such as non-target biomolecules and serum [12,13,14,15]. Therefore, several precise and sensitive detection methods have been developed to overcome these conditions. Among these detection methods, a fluorescent signal is an excellent and widely used technique for recognizing nucleic acids [16,17,18,19]. 

The fluorescence-based analytical methods provide precise, sensitive, and reproducible quantification of cancer-related biomarkers as well as nucleic acids. In addition, changes in the fluorescent signals by the biological event, such as DNA hybridization, can be easily observed. Based on these advantages, fluorescence-based detection methods have been widely utilized in typical biological experiments, including immunostaining and polymerase chain reaction (PCR). Over the past decades, fluorescence-based systems have been improved by integrating several nanomaterials, which translate the recognition of the interaction into the changes in fluorescence wavelength and intensity, such as fluorescence resonance energy transfer (FRET) and metal-enhanced fluorescence (MEF) effects, making these systems excellent platforms for nucleic acid–sensing for cancer diagnosis [19,20,21,22,23]. In this review, we describe the current state-of-the-art developments in nanomaterial-based fluorescent biosensors, with a focus on FRET and MEF, for quantification of diverse nucleic acids, including DNA and RNA, to diagnose cancers precisely and sensitively. We believe that the topics described and discussed in this review can provide practical information and a brief insight into the current status and prospect of developing a nanotechnology-based fluorescent nucleic acid–sensing system for biomedical applications. 

## 2. Nucleic Acid Targets

### 2.1. Genomic DNA (gDNA)

gDNA refers to the cellular DNA component that houses the biological information of the cell and can be passed on to the next generation. The genomic analysis provides information about the structural and molecular changes in DNA as well as the changes in gene expression. Analysis of the epigenetic changes in DNA can help identify new drug targets for cancer diagnosis and treatment [24]. Among the reported drug targets, 5-hydroxymethylcytosine (5-hmc) is downregulated in several types of cancer cells. This observation indicates that changes in the cellular level of 5-hmC can be used for cancer diagnosis. Therefore, high-performance liquid chromatography (HPLC) [25], fluorescence [26], electrochemistry [27,28], and electrochemiluminescence (ECL) [29] methods have been developed for cancer diagnosis.

### 2.2. Other DNAs

Cell-free DNA (cfDNA) refers to any DNA outside a cell. DNA methylation is an important epigenetic modification strongly implicated in the physiological regulation of gene expression. The DNA methylation patterns in cancer tissues differ from those in healthy tissues, irrespective of the tissue origin of cancer cells. This difference enables distinguishing cancer tissues from healthy tissues. In addition, DNA methylation patterns can be utilized for early cancer diagnosis, cancer-specific genetic testing, and cancer treatment [30,31,32]. The cfDNA in the bloodstream is in part caused by tumor-specific mutations, and this cfDNA sub-population is called circulating tumor DNA (ctDNA). Because ctDNA is present in the bloodstream, it is a potentially important biomarker for the early detection of cancer. However, the concentration of ctDNA in the bloodstream of a cancer patient is incredibly low, hampering a quick and accurate diagnosis of cancer. Therefore, studies on fast-responsive and ultrasensitive biosensors have been undertaken [33,34].

### 2.3. Messenger RNA (mRNA)

mRNA is a single-stranded molecule of RNA that is complementary to the genetic sequence of a gene and has been commonly used as a biomarker for early detection and treatment of cancer due to its humoral stability and biological regulatory function. It is usually quantitatively detected using quantitative reverse transcription-polymerase chain reaction (qRT-PCR) [35,36,37]. Although qRT-PCR is a very sensitive method, it is time-consuming and requires heavy instruments for the associated thermocycling reactions. Therefore, isothermal gene amplification [36,38], ECL [39], fluorescence [40,41], electrochemistry [42,43], and surface plasmon resonance (SPR) [44] have been developed as an alternative to PCR for mRNA detection.

### 2.4. Non-Coding RNA (ncRNA)

An ncRNA is a functional RNA molecule that is not translated into a protein. These RNAs can be classified as small RNAs (sRNAs) (19–31 nucleotides) or long ncRNAs (>200 nucleotides) [45]. MicroRNAs (miRNAs) of the sRNA families are known to be directly involved in gene expression. The expression of various miRNAs is dysregulated in human cancers through a variety of mechanisms [46,47]. Among such miRNAs, miRNA-21 is upregulated in colorectal cancer and induces cellular invasion, apoptosis, and drug resistance [48]. In addition, miRNA-203 has been observed to promote the carcinogenic transformation of cells and cancer cell proliferation. This miRNA is upregulated in breast cancer [49], whereas miRNA-330 is downregulated in prostate cancer and lung cancer cells [50]. Various miRNA detection platforms based on fluorescence [51], electrochemistry [52], ECL [53], or SPR [54] have been developed.

## 3. FRET-Based Nucleic Acid Biosensors for Cancer Diagnosis

### 3.1. FRET

**Figure 1:**
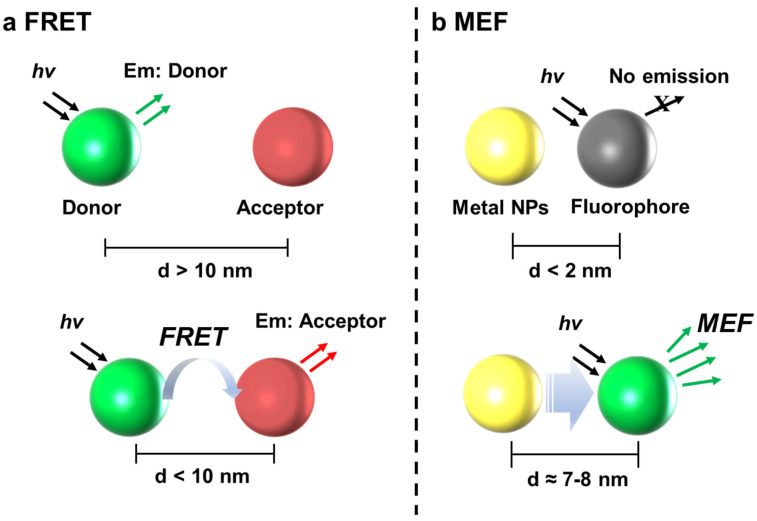
The simple mechanism of the (**a**) FRET and (**b**) MEF phenomena. FRET is induced when the distance between donor and acceptor is <10 nm. In the case of MEF, metal NPs can enhance the fluorescence intensity of fluorophore when the distance is around 7~8 nm.

FRET is a distance-dependent energy transfer process between two fluorophores, one of which acts as an energy donor fluorophore and the other as an acceptor. Thus, FRET can be used to measure the nanoscale distance between two interacting biomolecules (Figure 1a) [55,56,57,58]. The proximity between the donor and acceptor should be <10 nm, and for such a single fluorophore pair, the efficiency of FRET is inversely proportional to the sixth power of the distance between the donor and acceptor, so FRET changes the distance between donor and acceptor. This highly sensitive technique has been widely used as a powerful tool to study the intermolecular distance between fluorophores and molecular conformational changes [59,60,61]. Based on this principle, FRET is used for the detection and quantification of nucleic acids and proteins extracted from blood. However, because the concentrations of nucleic acids, including RNA and DNA, in the blood of cancer patients are extremely low, an ultrasensitive and accurate detection method is required. The integration of nanomaterials for ultrasensitivity and high specificity has improved FRET performance in the detection of DNA and RNA molecules. There are many studies on FRET-based nanobiosensors for nucleic acid detection (Table 1).

### 3.2. FRET-Based Biosensors for Detection of DNA Targets

**Figure 2:**
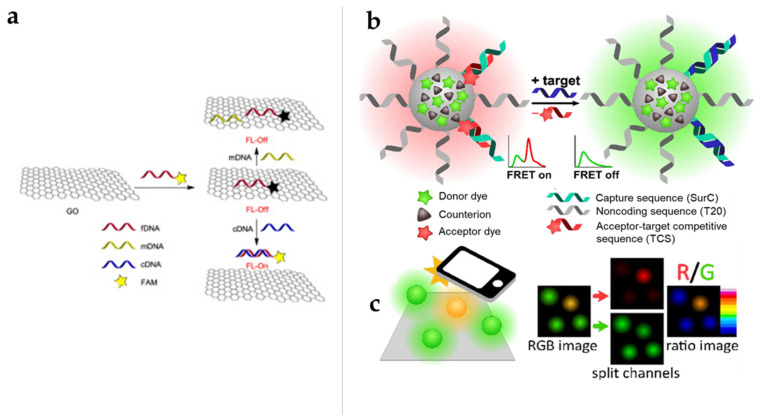
DNA target biosensor using nanomaterial-based FRET. (**a**) Schematic representation of the detection of a deletion mutation in exon 19 of the EFGR gene on a graphene oxide surface [68]. (**b**) Schematic diagram of the FRET-based RGB detection of DNA as a cancer biomarker by using a smartphone. This figure is reproduced from [64] (© 2016 Elsevier B.V.); Green emission from the nanoprobe (NP) surface transfers some of the excitation energy to the FRET receptor on the NP surface and, thereby, causes the NP to fluoresce in yellow–orange. (**c**) Smartphone-based data analysis. Green and red RGB images are taken, and intensity ratios between the channels are evaluated. This figure is reproduced from [66] (© 2020 Elsevier B.V.).

DNA detection at single-molecule sensitivity is the ultimate goal in biosensing and cancer diagnosis. Among the many techniques proposed for DNA detection, nanotechnology has shown great potential. Two dimensional (2D) nanomaterials with outstanding electronic and optical properties, such as broad absorbance, large surface area, and easily functionalized surface sites, can be utilized as acceptors or quenchers in FRET sensors [62,71,72]. To date, various nanomaterials, such as Au nanoparticles (NPs) [62], MoS_2_ [73], graphene oxide (GO) [63,64], and MXenes [66], have been developed for the fluorescence detection of DNA for cancer diagnoses. Eftekhari-Sis et al. developed a nanobiosensor based on graphene oxide (GO) and 5-carboxy fluorescein (FAM)-labeled DNA for the detection of a deletion mutation in exon 19 of the EGFR gene (Figure 2a) [65]. This system can detect a target DNA in a small amount of sample solution in 10 min with a very low detection limit of 25 fmol/µL. Dhenadhayalan et al. reported an ultrasensitive system based on molybdenum series (MoO_3_, MoS_2_, and MoSe_2_) of 2D nanosheets (NSs) for the detection of a prostate-specific antigen (PSA) as a diagnostic biomarker of prostate cancer [63]. The detection was achieved with 13 pM of MoO_3_ NSs, whereas MoS_2_ and MoSe_2_ NSs showed detection limits of 72 and 157 pM, respectively, among which the MoO_3_ sensor system showed a fast fluorescence response within 2 min. Severi et al. developed a FRET-based DNA nanoprobe for detecting DNA by using a smartphone camera [66]. The green-emitting NPs are based on rhodamine 110, and the 6G dye is paired with a massive hydrophobic counterion, which is a DNA cancer marker targeting polymer NPs functionalized with red-emitting oligonucleotides and the FRET receptor ATTO647N (survivin) (Figure 2b,c). Using a smartphone, survivin detection can easily detect nanoprobe responses at the 10 pM detection limits. 

### 3.3. FRET-Based Biosensors for Detection of RNA Targets

**Figure 3:**
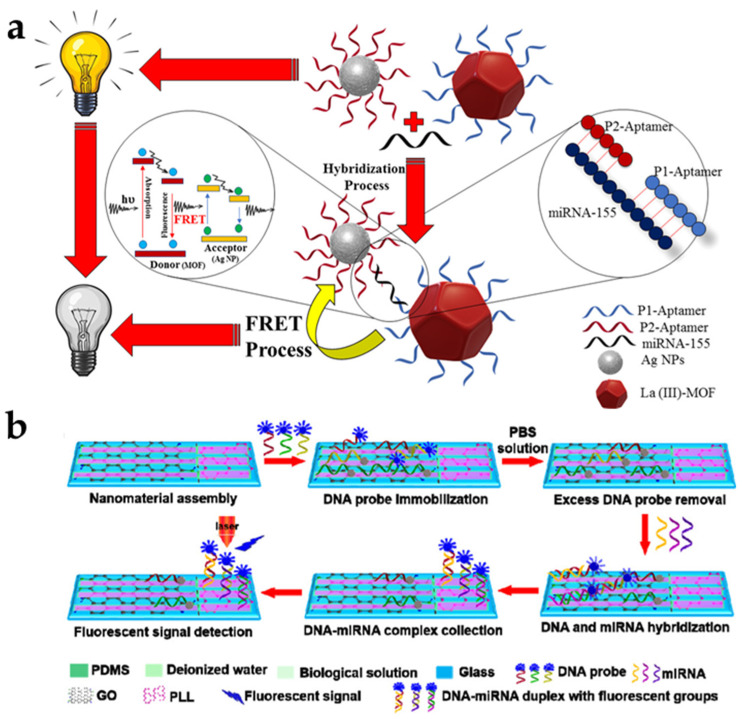
RNA target biosensor using nanomaterial-based FRET. (**a**) Schematic diagram of the FRET-based detection of miRNA-155 as a cancer biomarker by using La (III) metal–organic MOF and AgNPs. This figure is reproduced from [69] (© 2021 American Chemical Society); (**b**) Schematic illustration of the multiplex miRNA-sensing principle of microfluidic biochips via single-stranded probe/DNA–miRNA duplexes and PLL-assembled detection microchambers. This figure is reproduced from [70] (© 2021 American Chemical Society).

To realize low-level RNA detection in early cancer diagnosis and biological research, ultrasensitive, highly accurate, and fast RNA detection platforms have been developed. Among them, the use of a nanomaterial as a fluorescence quencher that effectively quenches the fluorescence of a fluorophore-conjugated probe via FRET is a widely used strategy. This technique offers advantages due to its large surface area and unique optical properties. Wang et al. reported the use of Ti_3_C_2_ nanosheets, a representative MXene, as biosensors for miRNA-21 detection [67]. Poly (acrylic acid) (PAA) decoration not only stabilized and increased the dispersion of the Ti_3_C_2_ nanosheets but also increased the number of covalent bonding sites for the subsequent surface DFNA functionalization. The detection limit of this strategy is 0.8 nM. Oudeng et al. developed Folic acid (FA)-functionalized MoS_2_ nanosheets immobilized with a fluorescently labeled single-stranded DNA probe (ssDNA–MoS_2_–PEG–FA) to detect miRNA-21, which is highly expressed in lung and pancreatic cancers [68]. In this system, the FA part, conjugated via an LA-PEG linker, protects the ssDNA probe and thus improves the cancer cell targeting and internalization processes. A higher binding force between the target miRNA-21 and the ssDNA probe was enhanced through the improved internalization process due to the hybridization of the endogenous miRNA and FA-functionalized MoS_2_ nanosheets (FAM)-ssDNA, and a fast fluorescence signal was confirmed due to the separation of the dye-labeled ssDNA probe from the MoS_2_ nanosheets. Afzalinia et al. used an La (III) metal–organic framework (MOF) and silver NPs (AgNPs) as the energy donor–acceptor pair in fluorescence quenching to detect the atypically expressed miRNA-155 in patients with breast or lung cancer [69]. The use of MOFs with large surface area significantly improved the miRNA detection performance by increasing the number of aptamers attached to the probe surface. This system can detect as low as 0.04 ppb (ng/mL) or 5.5 fM miRNA-155 (Figure 3a). Chu et al. developed a microfluidic biochip–based system that has an extremely low detection limit (0.146 aM) and the capacity to simultaneously detect up to 20 miRNAs in 35 min from an exceedingly small amount of sample solution [70]. In this system, glass substrates are locally assembled with GO and PLL in the reaction and detection chambers, respectively. The GO immobilizes a FAM-labeled DNA probe, and fluorescence is extinguished when the DNA probe is immobilized on the GO. PLL adsorbs both ssDNA and DNA–miRNA complexes without fluorescence quenching and collects the DNA–miRNA complexes after the target miRNA reacts with the DNA probe (Figure 3b). 

## 4. MEF-Based Nucleic Acid Biosensors for Cancer Diagnosis

### 4.1. MEF 

MEF has attracted much interest in both fundamental studies and sensing applications. Fluorescent intensities of fluorophores and fluorescent NPs could be expressively amplified due to their interactions with NPs based on noble metals, such as Au, Ag, Cu, and Pt [74,75,76]. The electromagnetic effect on the surface of the noble metal provides a significantly enhanced electric field, which enhances the excitation efficiency and increases the radiative decay rates of the fluorescent materials placed at the gap region. Consequently, the fluorescent intensities are increased. This MEF effect originates from the plasmon-coupling between the noble metal and fluorophores (Figure 1b). This fluorescence enhancement depends on the morphology and composition of the metal NPs, the distance between the metal NPs and fluorophores, and the absorption/emission spectral overlap between the metal NPs and fluorophores. Therefore, it is very important to design noble NPs with appropriated plasmonic properties to induce significant fluorescent enhancement. For the MEF effect, most of the fluorescent materials, including organic fluorophores, quantum dots, carbon dots, and upconversion NP, can be utilized coupled with noble NPs [77,78,79,80]. In addition, the MEF process simultaneously enhances the photostability and sensitivity as well as the intensities. Recently, MEF-based nanobiosensors have been developed to improve the detection sensitivity for target biomarkers, including nucleic acids and proteins, to the level of ultra-low concentrations [74,81,82]. The presence of fluorescent materials near the noble metal NPs increases the rate of excitation and emission by inducing the fluorophores to assume additional electron configurations. Based on this phenomenon, the MEF-based nanobiosensor consists of an optical transducer (fluorescent materials), and a signal amplifier (noble NPs) enables the construction of sensors simpler than those based on conventional fluorescence, which necessitates complicated steps to increase the detection sensitivity. In this regard, MEF is a promising tool for generating effective biosensors. Nucleic acid biomarkers have been known as excellent targets to diagnose several diseases, including cancer, due to the specific recognition of the complementary sequences. In addition, capture materials can be easily functionalized using various fluorophores and noble metal NPs. In this section, we discussed the currently developed MEF-based nanobiosensors for the detection of genetic materials to diagnose cancer. There are many studies on MEF-based nanobiosensors for nucleic acid detection (Table 2).

### 4.2. MEF-Based Biosensors for Detection of DNA Targets

**Figure 4:**
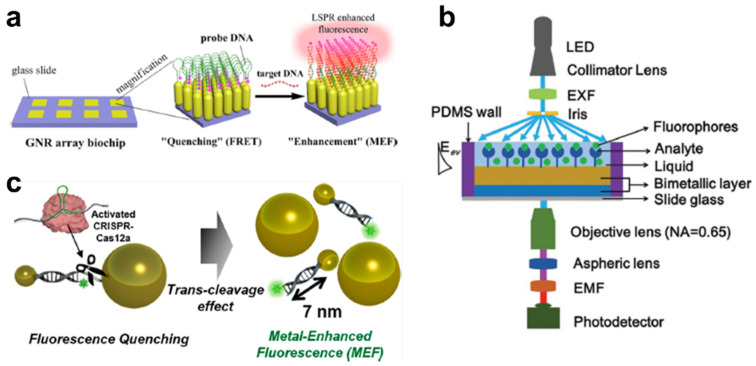
MEF-based nanobiosensor for DNA detection. (**a**) Schematic diagram of vertical Au-nanorod array for MEF-assisted DNA detection. This figure is reproduced from [86] (© 2017 American Chemical Society); (**b**) The straight (transmission) geometry-based bimetallic biosensing platform for the MEF-assisted DNA-detection system. This figure is reproduced from [87] (© 2018 John Wiley & Sons); (**c**) Schematic illustration of the CRISPR-Cas12a–based fluorescent biosensor for detection of DNA via a MEF using DNA-functionalized AuNPs without nucleic acid amplification. This figure is reproduced from [92] (© 2021 American Chemical Society).

Among the various DNA analytical methods, DNA microarray technologies have been widely used for disease diagnosis and DNA re-sequencing. For the detection of a target DNA, fluorescent materials are applied to translate the hybridization into a fluorescent signal. However, the sensitivity of fluorescence imaging is relatively low because the short half-lives of fluorescent probes restrict the photon intensity. Therefore, enhancement of the fluorescent signal is essential to sensitively quantitate the DNA target for precise and early diagnosis. An Ag-nanostructure was one of the remarkable substrates used for inducing MEF in a sensitive DNA microarray–based system [83]. The Ag-nanostructures on the films provided a 28-time intense signal from the near-infrared dye Cy5, compared with that obtained using glass substrates. In the case of DNA hybridization, its sensitivity increased 10-fold for Cy5 and 2.5-fold for Cy3. Ji et al. synthesized a zigzag-shaped Ag-nanorod to improve the MEF effect and found the optimal folding number to be seven, whose scattering intensity was maximized [84]. For the verification of the enhancement factor, neutravidin-coated fluorescent nanospheres were applied onto the biotinylated Au-nanorods, whereby a 14-fold increase in the emission intensity was obtained. This condition also enabled highly sensitive detection of DNA, as low as 0.01 pM concentration. Mei et al. developed a monolayered Au-nanorod in a highly ordered form for the induction of the localized surface plasmon resonance effect (LSPR). This system involves hot spots generated between the neighboring particle tips in the nanoarray [85]. Through this plasmonic effect, MEF was found to be dependent on the excitation and emission wavelengths of the fluorophore, greater than 600 nm. Using this phenomenon, a sensitive MEF-based biosensor was developed by integrating the molecular beacon detection technique in a chip-based format for ultrasensitive DNA analysis, as low as 1 pM. Badshah et al. achieved sensitive detection of DNA by using Ag vertical nanorod (VNR) arrays fabricated via the glancing angle deposition (GLAD) method (Figure 4a) [86]. A homogeneous VNR nanoarray generated at a specific incident angle (θ = 85°) with substrate rotation produced a better MEF effect than slanted nanorods (SNRs). In the case of the size, a maximum enhancement factor of approximately 200 times was attained on the NR substrate with a size of 500 nm, compared with the commercially available amine slide. Tran et al. used a bimetallic structure to maximize the MEF effect (Figure 4b) [87]. This bimetallic condition involved Au and Ag at a thickness of 2 and 50 nm, respectively. Under these conditions, a better local field was obtained, compared with that obtained using monometallic structures with an SYBR Green-conjugated double-stranded DNA. Using this nanosubstrate, the target DNA, amplified using polymerase chain reaction (PCR), was successfully quantitated at a concentration as low as 400 fg/μL and with reduced photobleaching. The authors claimed that this bimetallic nanosubstrate could provide a highly reproducible and sensitive platform for fluorescent-based DNA detection from a small sample volume in multiplexed diagnosis. 

In addition to the microarray system, solution-based DNA analytical methods have been widely developed using the nanomaterial-assisted MEF effect. Zhou et al. developed an AgNP-mediated DNA-detection system by using the MEF effect on the fluorophore (FAM) [88]. Because the AgNP modified two different DNAs, namely the capture DNA and barcode DNA, with FAM, this system could detect the target DNA and enhance the fluorescent signals simultaneously. The target DNA could induce the magnetic NP and AgNP to assume a sandwich conformation due to the hybridization reaction. After the magnetic separation method, DNA could be measured at a concentration as low as 1 pM and with high sensitivity and simplicity. Gu et al. utilized the hybrid magnetic Au-nanoclusters to induce the MEF effect via the hybridization of the target DNA [89]. To form a unique magnetic Au-nanocluster, amine-functionalized Fe_3_O_4_ NPs were applied to the AuNP to couple with the amine–Au binding reaction. The fluorescein isothiocyanate (FITC)-tagged capture DNA was functionalized on the AuNPs for the detection of the target DNA, whereby a significant distance-dependent MEF effect between the AuNP and FITC was obtained if the target DNA was bound to the capture DNA. In this study, the suitable distance for the MEF effect was estimated at approximately 4 nm, and the enhancement factor was approximately 10. Wu et al. developed a novel fluorescent nanoprobe consisting of a metal–organic framework (MOF), which is a highly ordered nanoporous structure with thermal stability [90]. The advantage of the MOF is a higher surface-to-volume ratio than the typical NPs because of their high porosity. Thus, a high quantity of molecular beacon-structured DNA could be attached from the core to the surface of the MOF. Additionally, the target DNA induced a fluorescent enhancement effect by displacing the fluorescent dye from the surface of the MOF. This MOF-based MEF system could quantitate a target DNA with a detection limit of 20 fM and with high selectivity. Zhu et al. identified the optimal size and shape of the metal NP for sensitive MEF-based DNA detection [91]. The AuNP-conjugated Cyanine 5 (Cy5) was quenched by the energy transfer to the AuNP. After the complementary binding of the target DNA with the capture DNA on the AuNP and another DNA on the Au-nanorod, AuNP, or Au@AgNP, the fluorescent signals could be enhanced by the MEF effect. Of the three different NPs, Au@AgNP showed the most enhanced result, which was approximately 100 times that of the quenched state. Using this coupled nanostructure, the target DNA could be detected at a concentration as low as 3.1 pM and without the pulse-positive signal of the single-mismatched DNA. Our group has developed a DNA-detection system by changing the quenched MEF state through the modulation of the target DNA concentration (Figure 4c) [92]. The 20 nm and 60 nm AuNPs connected by the double-stranded and single-stranded DNA. If the CRISPR-Cas12a was activated by the target DNA, it randomly cleaved the ambient single-stranded DNA, and the 20 nm AuNPs apart from another AuNP. This phenomenon induced the MEF effect between the fluorophore and the 20 nm AuNP, and the target DNA was sensitively quantitated within 30 min at a concentration ranging from 1 fM to 100 pM. Thus, metal NPs can assist the sensitive detection of DNA through the metal-based fluorescence enhancement effect in both DNA microarrays and solution-based systems. 

### 4.3. MEF-Based Biosensors for Detection of RNA Targets

**Figure 5:**
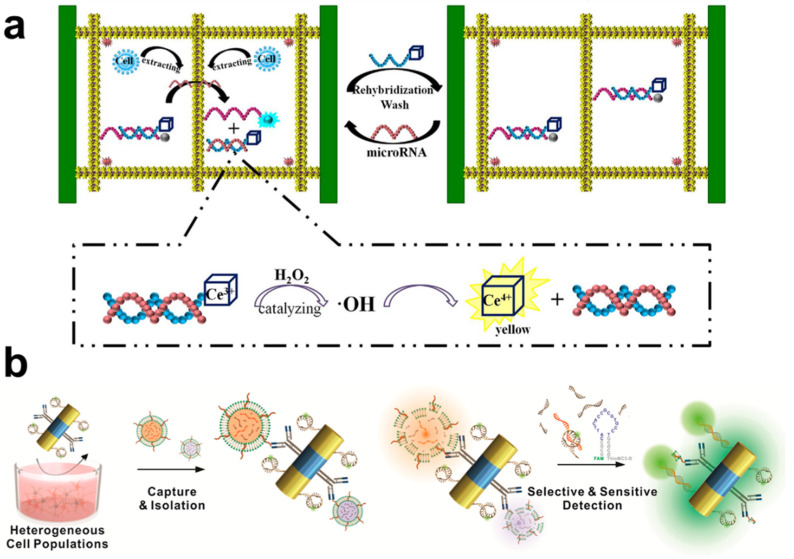
MEF-based RNA nanobiosensors. (**a**) Schematic diagram of the Au-based triangular nanoprism biosensor for detection of miRNAs via the SERS and MEF methods. This figure is reproduced from [94] (© 2017 Elsevier B.V.); (**b**) Schematic illustration of exosomal miRNA detection by using multifunctional Au-Ni-Au nanorods via the MEF effect. This figure is reproduced from [98] (© 2019 American Chemical Society).

As mentioned before, RNA molecules are also potential biomarkers for the diagnosis of several diseases because of the potential pathophysiological roles of these molecules. Therefore, there have been several attempts to quantitate specific RNAs by integrating the nanomaterial-assisted MEF effect. The first attempt was approximately 15 years ago and consisted of a silver-island film on a glass substrate [93]. In this system, a 484-mer RNA is attached to the silver-island film via two 15-mer complementary RNAs as a sandwich structure. A TAMRA-tagged 15-mer RNA emits enhanced fluorescence and can successfully detect a low concentration of RNA (~25 fM). Recently, Liang et al. developed the flower-like silver (FLS)-enhanced fluorescence biosensor for ultrasensitive detection of multiple miRNAs (Figure 5a) [94]. To simplify the detection process, the authors applied the multi-channel microfluidic paper-based analytical devices (μPADs) to the MEF-based biosensing system. The carbon dot and nanoceria (CeO_2_) were conjugated to each other before the binding of the target miRNA, and the fluorescent signal was quenched due to the CeO_2_. After hybridization of the target miRNA to the carbon dot-modified capture DNA, the signal emitted by the carbon dot was enhanced, and the target miRNA, at the concentration of as low as 0.03 pM, could be quantitated. Simultaneously, CeO_2_ showed the catalytic activity on the H_2_O_2_, which was one of the reactive oxygen species. This platform could provide the real-time monitoring of miRNAs and H_2_O_2_ with high sensitivity and selectivity. Wang et al. have developed a microfluidic-based miRNA detection platform, which consisted of surface-enhanced Raman scattering (SERS) and a MEF-inducing nanosystem [95]. AgNPs were immobilized on a glass substrate, forming SERS and MEF substrates simultaneously with a Raman and fluorescent dye (FAM)-tagged molecular beacon. When the target miRNA hybridized with the molecular beacon, it increased the distance between the FAM and AgNP, and the fluorescent intensity of the FAM increased, whereas the SERS signal decreased. By combining the reverse changes, the target miRNA, at a concentration of as low as 1 pM, could be detected with reduced reaction duration and complexity. This dual analytical strategy can circumvent each disadvantage and stand out for each advantage. Masterson et al. synthesized Au-based triangular nanoprisms and applied them to a nanoplasmonic biosensing platform for sensitive miRNA detection [96]. This triangular nanostructure facilitated the induction of the plasmonic effect, thereby inducing both SERS and MEF effects. By using this system, the oncogenic miRNAs (miR-10b and miR-96) in the serum of a patient were directly quantitated with high sensitivity (at the sub-fg/μL level). Importantly, the dual-sensing nature of this system prevents false positive and negative responses.

Solution-based sensitive detection of RNA has also been studied using several detection strategies with metal NPs. Lu et al. developed miRNA biosensors via a fluorescence enhancement strategy with biotin-functionalized lanthanide NPs as signal enhancers [97]. A surface-modified molecular beacon could capture the target miRNA, and the biotinylated detection probe hybridized with the miRNA as a sandwich structure. The enhanced fluorescent signal from the lanthanide NPs provides sensitive detection, ranging from 10 fM to 100 pM, with a detection limit as low as 1.38 fM, which is three orders of magnitude better than the typical fluorescent probes. Our research group has detected exosomal miRNAs by using MEF with a triblock nanorod, which consisted of Au-Ni-Au (Figure 5b) [98]. FAM-tagged molecular beacon modified on the surface of Au with the quenched state. Once the antibody on the Ni surface captures the exosomes, they are separated by the magnetic force, and any ejected exosomal miRNA binds to the molecular beacon specifically. The unfolded molecular beacon can emit an enhanced fluorescence signal due to the plasmonic effect of the Au-nanorod. Using this particle-based MEF sensing system, miR-124, which is closely related to neuronal differentiation, has been measured in a highly sensitive manner, as low as 1 pM. This method could also characterize a heterogeneous population of neural cells, including neurons and astrocytes, in an in vitro cell culture model and ex vivo rodent model. We anticipate that this multi-segmented MEF-based exosomal miRNA biosensing platform has a prodigious potential to diagnose several cancers and investigate intra- and extracellular communications with high sensitivity. 

## 5. Outlook and Conclusions

Nanomaterials can enhance the fluorescent signal via MEF, and ultrasensitive detection of target genetic materials has been achieved for the early diagnosis of several diseases and observation of cell–cell interactions. However, there is still room to improve for precise, accurate, and early diagnosis. For a precise and accurate diagnosis, measurement of only one nucleic acid biomarker is insufficient, even though the result of such detection shows high reliability. To solve this problem, additional biomarkers should be identified. A multi-analyte detection system can provide a precise diagnosis. For example, quantitation of a single miRNA cannot indicate the exact state of cancer. However, a multi-miRNA analysis can provide more information about the cancers [99,100,101]. In this perspective, nanomaterial-based fluorescent biosensors should be developed to quantitate multiple nucleic acids simultaneously. Fluorescent nanomaterials, including upconversion NPs or quantum dots, can be applied to the multi-detection platform by virtue of their specific emission peaks and higher quantum yield than typical organic dyes. For early diagnosis, a sensitive detection system should be developed to measure infinitesimal changes in the amount of nucleic acid biomarkers. As mentioned previously, nanomaterials can improve the sensitivity as well as selectivity and shorten the assay time. Furthermore, fluorescent-based analytical methods as well as electrochemical, electrical, and other optical methods have also been applied to quantitate nucleic acid biomarkers at a highly sensitive level. Such nanomaterial-based biosensors should be integrated into user-friendly devices, such as a disposable, wearable, or smartphone-based point-of-care testing system. These integrated systems will enable the treatment of the disease quickly and with the proper treatment methods and drugs. 

In conclusion, we reviewed the recent progress in the use of nanotechnology-based FRET and MEF biosensors for the detection of nucleic acid biomarkers. Compared with conventional fluorescence biosensors, FRET and MEF exhibit superb performances, such as intensive emission spectra for sensitive detection. As well as nucleic acid biomarkers, other biomolecules, such as enzymes, have been measured using NP-assisted FRET and MEF. In addition, these analytical methods can provide the intuitive result upon integration to other platforms, such as disposable or smartphone-based systems. As a result, FRET and MEF biosensing platforms for sensitive, selective, simple, and rapid detection will be further developed using various nanotechnologies to improve the current detection performances. The advanced fluorescence-based sensing platform with superior properties of nanomaterials will provide personalized therapies with precise and detailed diagnostic results to increase the recovery rate in several diseases, including cancers.

## Figures and Tables

**Table 1:** Comparison of FRET-based nanobiosensors for nucleic acid detection.

Feature	Donor/Acceptor	Wavelengths (Ex,Em)	Target	Required Time	Detection Limit	Ref
FAM-labeled DNA probe adsorption on AuNPs via thiol-modified probes; When hybridizing with target DNA, fluorescence occurrence due to reduced interaction and increased distance.	AuNPs/FAM-labeled DNA probe	Ex: 545 nm Em: 580 nm	methylated DNA	>2 h	2.2 pM	[62]
Aggregation-induced release (AIE) molecules and cDNA adsorption on GO via π-π stacking; Increased distance by reduced interaction when hybridizing with target DNA eliminates quenching effect	GO/AIE labeled DNA probe	Ex: 370 nm Em: 465 nm	DNA	3 min	3.1 pM	[63]
FAM-labeled DNA adsorption on GO via π-π stacking; FAM-labeled ssDNA desorption and fluorescence recovery from GO through addition of target DNA	GO/FAM-labeled DNA probe	Ex: 488 nm Em: 520 nm	Exon 10 (EGFR gene)	10 min	35 fmol/μL	[64]
FAM-labeled DNA adsorption on Ti_3_C_2_ NSs via π-π stacking; Detection of PCR-amplified HPV-18 DNA from cervical scrape samples	Ti_3_C_2_ NSs/FAM-DNA probe	Ex: 495 nm Em: 520 nm	HPV-18	5 min	100 pM	[65]
Target DNA detection by Rho 110 and 6G-PMMA using a smartphone RGB camera using a two-color fluorescence microscope; Prevention dye self-quenching of polymer nanoparticles encapsulating green fluorescent donor (Rho 110 and 6G) and hydrophobic counterions (ATTO647N) as acceptors	Rho 110/6G-PMMA/ATTO647N	Ex: 488 nm Em: 662 nm	DNA	3 h	10 fM	[66]
Covalent binding of TAMRA-labeled MUC1 aptamer and TAMRA-labeled miR-21 to the surface of chimeric DNA-functionalized Ti_3_C_2_; Red and green fluorescence recovery by nanoprobe and target MUC1, miR-21 hybridization.	Functionalized Ti_3_C_2_/TAMRA- miR-21, TAMRA-MUC1	Ex: 488 nm Em: 525 nm	miR-21 MUC1	2 h	0.8 nM	[67]
Isolation and green fluorescence recovery of dye-labeled ssDNA on the surface of MoS_2_ nanosheets due to hybridization between the probe and the target miR.	MoS_2_/FAM-miR 21 probe	Ex: 488 nm Em: 520 nm	miR-21	> 2 h	10–50 nM	[68]
Detection of UV-vis and gel electrophoresis from 1-aptamers attached to the probe surface using high surface area MOFs.	La (III) MOFs/AgNPs	Ex: 220 nm Em: 430 nm	miR-155	45 min	5.5 fM	[69]
FAM-labeled DNA adsorption on GO via π-π stacking; Collection and detection of DNA-miR complexes after the target miRNA hybridization with the probe and detaches from the reaction channel.	GO/FAM-labeled DNA probes	Ex: 488 nm Em: 520 nm	miR-125, miR-126, miR-191, miR-155, miR-21,	35 min	0.146 aM	[70]

**Table 2:** Comparison of MEF-based nanobiosensors for nucleic acid detection.

Detection Strategy	Fluorescent Dye/Enhancer	Wavelengths (Ex, Em)	Target	Required Time	Detection Limit	Ref
Detection of DNA by using engineered substrates to increase the emissivity of the fluorophore (proximity of surface plasmons (<100 Å))	Cy3, Cy5/Ag-nanostructure	Ex: 549 nm, 646 nm Em: 562 nm, 663 nm	DNA	<3 h	-	[83]
By applying neutravidin-coated fluorescent nanospheres to biotinylated Au-nanorods, the signal enhancement under the fluorescence microscope using Ag zigzag nanorods (ZNR) arrays	Alexa 488/Ag zigzag nanorod	Ex: 488 nm Em: 525 nm	DNA	<3 h	0.01 pM	[84]
Induction of localized surface plasmon resonance effect (LSPR) of highly ordered monolayer Au-nanorods; Distance-dependent MEF effect between extinction and enhancement	Quasar670/Au-nanorod	Ex: 664 nm Em: 670 nm	ssDNA	<20 min	10 pM	[85]
Detection of DNA by using Ag vertical nanorod (VNR) arrays fabricated via the glancing angle deposition (GLAD) method	Cy5/Ag vertical nanorods	Ex: 635 nm Em: 670 nm	Kallikrein (KLK7)	>12 h	-	[86]
Bimetallic structures (containing Au and Ag 2 nm and 50 nm thick) are used to maximize the MEF effect; Detection of amplified target DNA using polymerase chain reaction (PCR).	SYBR Green I/Au-Ag	Ex: 470 nm Em: 535 nm	dsDNA	-	400 fg/μL	[87]
Fix with capture DNA of magnetic nanoparticles implanted with silver nanoparticles; After introduction of target DNA, sandwich structure formation by hybridization reaction and isolation of barcode DNA from silver nanoparticles and fluorescence enhancement	FAM/NMP-AgNP	Ex: 495 nm Em: 518 nm	DNA	>10 min	1 pM	[88]
Increase the signal-to-background ratio by using the high quenching efficiency of AuNPs; Distance-dependent MEF effect and high specificity of target fluorescein isothiocyanate-tagged DNA-HMNC and target DNA hybridization	FITC/Magnetic NP/nanogold clusters	Ex: 488 nm Em: 520 nm	DNA	>2 h	0.01 μM	[89]
Amine-functionalized Fe_3_O_4_ nanoparticles bind with amine-gold bonding reaction and form magnetic Au-nanoclusters; After binding of target DNA, distance-dependent MEF effect between AuNP and FITC	Flu, Rho 6G, Cy5/MOF-based nanoprobes	Ex: 494, 525, and 646 nm Em: 513 nm, 553 nm, 664 nm	DNA	<30 min	20 fM	[90]
Quenching of AuNPconjugated Cy5 by AuNP, complementary binding of target DNA with capture DNA on AuNP and other DNA on Au-nanorod, AuNP or Au@AgNP, and enhancement of fluorescence signal.	Au-NP-conjugated Cy5/Au@AgNP	Ex: 410 nm Em: 660 nm	DNA	>12 h	3.1 pM	[91]
Length optimization of dsDNA and attachment of Au-nanoparticles (AuNPs) to the surface; Activation of CRISPR-Cas12a complex by target cfDNA, degradation of AuNP and fluorophore ssDNA, and generation of red-violet fluorescence	FITC/DNA-functionalized AuNP	Ex: 490 nm Em: 520 nm	breast cancer gene-1 (BRCA-1)	<30 min	0.34 fM	[92]
Annealing of target RNA and DNA anchor probes tagged with silver nanoparticles and fluorophores on a solid surface and enhancement of fluorescence signal	TAMRA/AgNP	Ex: 532 nm Em: 585 nm	β-globin mRNA	30–60 min	25 fM	[93]
Fabrication of sandwich structure using 484-mer RNA and 15-mer complementary RNA attached to Flower-like silver film; Enhanced fluorescence emission of TAMRA-tagged 15-mer RNA; Shortening the spacing distance between N-CD and CeO_2_ and significantly quenching the fluorescence of N-CD; Fluorescence recovery of quenched probes in the presence of target miRNAs	(DNA_1_-NCDs)/Flower-like Ag	Ex: 390 nm Em: 462 nm	miR-210, miR-21	15 min	0.03 fM, 0.06 fM	[94]
FAM-MB formed a hairpin structure and the fluorescence reduction of 6-FAM and AgNPs; Upon target miRNA hybridization, increasing the distance between 6-FAM and AgNPs and Fluorescence enhancement	FAM-tagged MB/AgNRs	Ex: 488 nm Em: 520 nm	miR-21	60 min	1 pM	[95]
Fluorescent dye (FAM) tagging of chemically synthesized gold triangular nanoprisms (Au TNPs); When the target miRNA hybridizes with the molecular beacon, the distance between the FAM and AgNP increases and the fluorescence intensity of the FAM increases.	FAM/Au TNPs	Ex: 488 nm Em: 520 nm	miR-10b, miR-96	120 min	1.13 pM, 30 fM	[96]
Fluorescence enhancement using biotin-functionalized lanthanide nanoparticles as signal enhancers and capture of target miRNAs of surface-modified molecular labels; Detection of Biotin-NPs-probe hybridized with miR in a sandwich structure.	Biotin-NPs/Ln^3+^-nanoprobe	Ex: 340 nm Em: 617 nm	miR-21	>2 h	1.38 fM	[97]
The miR-124 specific MB (molecular beacon) attachment on magnetic plasmonic nanorods; Immunomagnetic isolation and enrichment of exosomes in cell culture medium collection, non-destructive analysis of exosome miR-124 expression.	FAM/plasmonic AuNRs	Ex: 490 nm Em: 520 nm	miR-124	30 min	1 pM	[98]

## Data Availability

Not applicable.
